# Should Symbionts Be Nice or Selfish? Antiviral Effects of Wolbachia Are Costly but Reproductive Parasitism Is Not

**DOI:** 10.1371/journal.ppat.1005021

**Published:** 2015-07-01

**Authors:** Julien Martinez, Suzan Ok, Sophie Smith, Kiana Snoeck, Jon P. Day, Francis M. Jiggins

**Affiliations:** Department of Genetics, University of Cambridge, Cambridge, United Kingdom; Monash University, AUSTRALIA

## Abstract

Symbionts can have mutualistic effects that increase their host’s fitness and/or parasitic effects that reduce it. Which of these strategies evolves depends in part on the balance of their costs and benefits to the symbiont. We have examined these questions in *Wolbachia*, a vertically transmitted endosymbiont of insects that can provide protection against viral infection and/or parasitically manipulate its hosts’ reproduction. Across multiple symbiont strains we find that the parasitic phenotype of cytoplasmic incompatibility and antiviral protection are uncorrelated. Strong antiviral protection is associated with substantial reductions in other fitness-related traits, whereas no such trade-off was detected for cytoplasmic incompatibility. The reason for this difference is likely that antiviral protection requires high symbiont densities but cytoplasmic incompatibility does not. These results are important for the use of *Wolbachia* to block dengue virus transmission by mosquitoes, as natural selection to reduce these costs may lead to reduced symbiont density and the loss of antiviral protection.

## Author Summary

Arthropods are commonly infected with heritable bacteria, and some of these symbionts can protect their hosts against infection and/or be reproductive parasites. Which of these traits evolves will depend on whether the trait is costly to the symbiont and the host. Using a panel of strains of the symbiont *Wolbachia* in the fruit fly *Drosophila simulans*, we found that the beneficial effect of antiviral protection and the parasitic phenotype of cytoplasmic incompatibility occur independently across the strains. We found that high antiviral protection is associated with high symbiont densities and strong reductions in other life-history traits affecting the fitness of both the symbiont and the host. In contrast cytoplasmic incompatibility did not induce costs on these traits. This trade-off between antiviral protection and other fitness components may select for reduced antiviral protection, which would endanger the long-term success of programs using *Wolbachia* to block the transmission of mosquito-borne viruses.

## Introduction

Heritable symbionts are frequent in insects and their evolutionary success relies on various strategies. By sharing a common route of transmission with their host’s genes, they benefit from increasing host fitness. Consequently, numerous endosymbiotic bacteria evolved towards mutualism, for example by complementing their host diet [1,2], increasing tolerance to environmental stresses [3] or protecting against natural enemies [4–9]. However, because most of these heritable bacteria are maternally-transmitted, the evolutionary interests of host and symbiont are not perfectly aligned since only females transmit the symbiont. This has led to many symbionts evolving selfish strategies that consist of parasitic manipulation of their host’s reproduction by inducing female-biased sex-ratios or cytoplasmic incompatibility (CI) [10]. CI is a sperm modification that results in embryonic mortality in crosses between uninfected females and males harboring the symbiont, thus giving a competitive advantage to infected females that can rescue the sperm modification. Mutualism and reproductive manipulation are not mutually exclusive, and some symbionts display both [11]. However, the balance between the benefits and costs of these extended phenotypes to the symbiont’s fitness, as well as the genetic correlations between them, will determine which of these strategies is favoured by natural selection.

*Wolbachia*, which are common maternally-transmitted bacterial symbionts of arthropods, can be both parasites and mutualists. *Wolbachia* has been shown to protect *Drosophila* and mosquitoes against several RNA viruses—including Dengue and Chikungunya viruses [7,9,12–15]. Some strains also protect insects against filarial nematodes [16], *Plasmodium* parasites [12,17,18] and pathogenic bacteria [19]. Although it is unclear how important antiviral protection is in nature and whether it is under strong selection, some protective *Wolbachia* strains are able to invade host populations while inducing no other known phenotypes [20,21]. In addition, *Wolbachia* has the ability to spread rapidly through insect populations by parasitically manipulating reproduction, in particular by CI [22]. This combination of traits makes *Wolbachia* an attractive tool for blocking disease transmission by mosquitoes, as CI allows it to spread through vector populations while its antiviral effects can prevent them from transmitting arboviruses [23,24].

Levels of both antiviral protection and CI may evolve rapidly. During the 20^th^ century in natural populations of *D*. *melanogaster* the *Wolbachia* strain *w*MelCS, which provides strong antiviral protection, was partially replaced by *w*Mel [25,26], which provides weaker protection [27]. In North American populations of *D*. *simulans*, field and experimental data suggest that the strain *w*Ri has evolved to produce weaker levels of CI within a few decades [28].

Efforts to use *Wolbachia* to block the transmission of viruses have focused largely on the mosquito *Aedes aegypti*, which is the primary vector of dengue virus. *Wolbachia* has been successfully introduced into two Australian populations of *Aedes aegypti* [29], and three years post-release it had reached a stable and high prevalence in the field despite having a negative effect on the fecundity of mosquitoes [30]. Both antiviral protection and levels of CI were maintained over time [30,31].

In the long-term, the presence of fitness costs is expected to select for both host genes and bacterial genes that reduce these costs [32]. In accordance with this prediction, the *Wolbachia* strain *w*Ri evolved from reducing the fecundity of the flies to increasing it within two decades in North American populations of *D*. *simulans* [33]. It is possible that the evolution of lower costs could be achieved by a decrease in bacterial densities, as costly *Wolbachia* tend to have high bacterial densities [27,34,35]. Since a high *Wolbachia* density may be required for the expression of both antiviral protection [14,27,34,36–38] and CI [35,39–42], the evolution of reduced *Wolbachia* density might translate into a correlated decrease in the ability to block arbovirus transmission and invade insect populations.

To investigate these questions, we used sixteen *Wolbachia* strains in a common host genetic background to measure the level of CI induced and effects on other fitness-related traits, and have tested for correlations between these traits and antiviral protection. Our results demonstrate that antiviral protection is independent of CI but that it is associated with reduction on other fitness components. Furthermore, this trade-off can be explained by the density of the bacteria in the somatic tissues of the insect. Overall, our study suggests that newly introduced *Wolbachia* infections may evolve towards weaker protection in the field.

## Results

**Fig 1:**
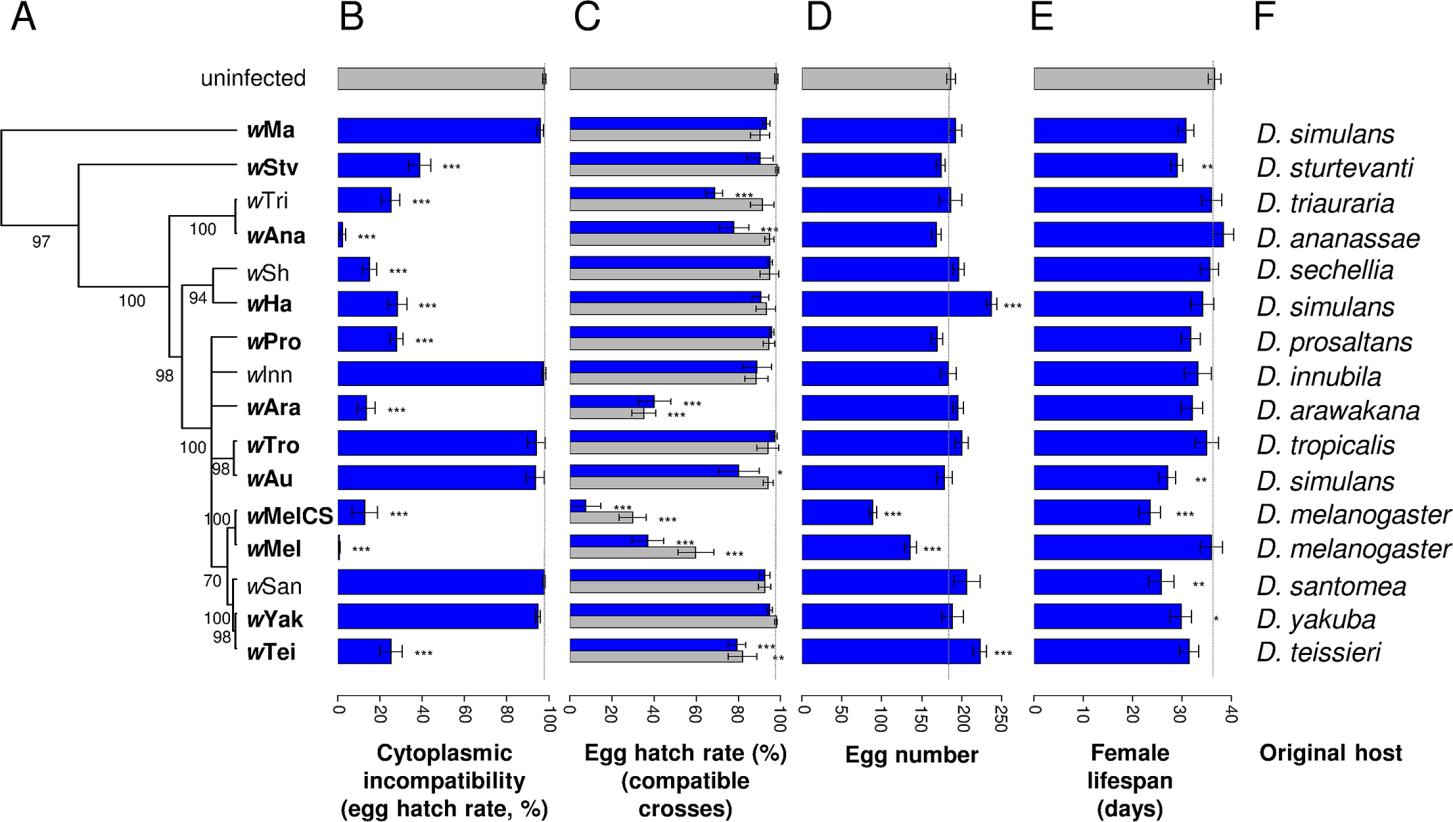
Phylogenetic distribution of CI levels and *Wolbachia* effects on egg hatch rates, fecundity and lifespan. (A) The phylogeny based on the MLST genes *16S rRNA*, *aspC*, *atpD*, *ftsZ*, *sucB*, *groEL*, *coxA* and *fbpA* was inferred using ClonalFrame v1.2 [43] as in [14]. Strains in bold conferred significant antiviral protection [14]. Branch labels represent posterior support values. Nodes with less than 50% support were collapsed. Branch lengths indicate relative time. (B) CI measured as egg hatch rates in crosses between uninfected females and *Wolbachia*-infected males. (C) Egg hatch rates in crosses between *Wolbachia*-infected females and *Wolbachia*-infected males (blue bars) or uninfected males (grey bars). (D) Fecundity of *Wolbachia*-infected females. (E) Lifespan of *Wolbachia*-infected females. Error bars are standard errors. *: significance relative to the *Wolbachia*-free line (Dunnett’s test; *: *P* < 0.05; **: *P* < 0.01; ***: *P* < 0.001). The dotted line indicates for each trait the mean value in the *Wolbachia*-free controls. (F) Original host species of the *Wolbachia* strains.

To compare multiple symbiont strains independent of host genetic effects, we used a panel of *Wolbachia* strains that had been transferred from different *Drosophila* species into a single inbred line of *D*. *simulans* (Fig 1F). To avoid effects of using an inbred fly line, we crossed these flies to a different inbred fly line and used the F1 progeny in our experiments. Vertical transmission rates were previously estimated and were 100% for all *Wolbachia* strains used in this study [14].

### Cytoplasmic incompatibility and antiviral protection are independent traits

Cytoplasmic incompatibility causes an excess of embryonic mortality in crosses between symbiont-infected males and uninfected females. Therefore, in order to measure levels of CI induced by different *Wolbachia* strains, we crossed infected males of each strain with uninfected females and counted the number of eggs that hatched (9,432 eggs from 380 females). There was a significant effect of *Wolbachia* (Deviance = 681.81; df = 16; *P* < 0.0001) with a clear division between 10 strains that induce CI and six that do not (Fig 1B). The strength of CI also varied among the 10 CI strains, ranging from just 0.5% of the eggs hatching in incompatible crosses involving the *w*Mel strain, to 38.7% of the eggs hatching with *w*Stv.

**Fig 2:**
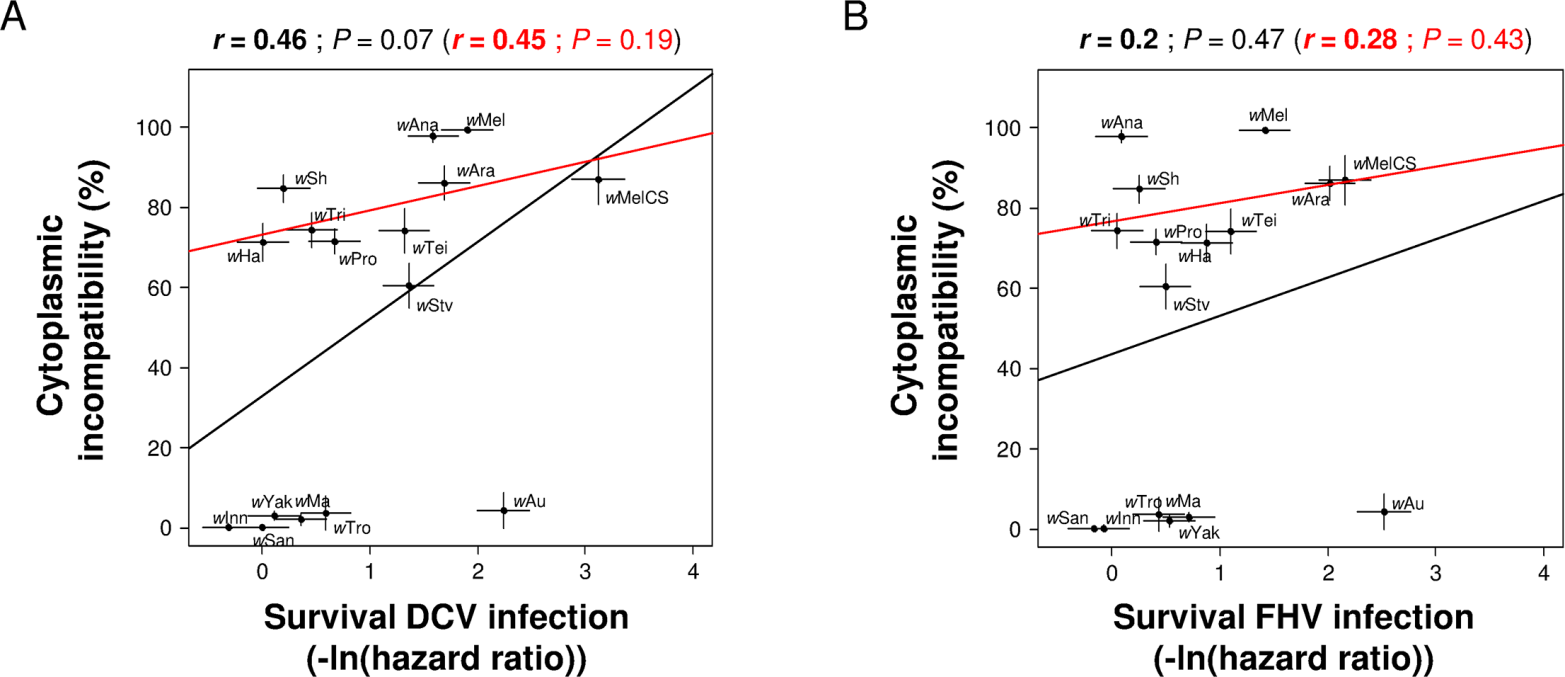
Correlation between CI and antiviral protection. Levels of CI estimated as the percentage of unhatched eggs relative to the mean hatch rate in crosses between uninfected females and uninfected males. Level of protection measured as survival in [14] upon infection with (A) DCV and (B) FHV (0 and positive values mean no difference and increase in survival compared to *Wolbachia*-free control respectively). Means and standard errors are shown. Solid lines show predicted values from linear regressions using all strains (black) or only CI-inducing strains (red). *r* is the Pearson’s correlation coefficient between traits.

We have previously shown that these strains provide varying levels of protection against the viruses DCV and FHV [14], and using this data we found that there was no correlation between CI and the antiviral effects of *Wolbachia*. This was the case regardless of which virus the flies are infected with or whether antiviral protection is measured in terms of increased survival (black line in Fig 2A and 2B) or reduced viral titre (black line in S1A and S1B Fig). This conclusion also holds if we only analyse the 10 strains that induce significant CI (red line in Fig 2A and 2B; S1A and S1B Fig). Since a decrease in hatch rate in incompatible crosses can be due not only to CI but also to an induced cost on male fertility, we also analysed the correlation between protection and levels of CI corrected for differences in male fertility (the hatch rates of infected females mated with infected males relative to hatch rates when mated with uninfected males). Similar to the uncorrected estimate, these corrected levels of CI did not show any significant correlation with antiviral protection, whether measured as survival after infection (Pearson’s correlation test: All strains: DCV: *P* = 0.28 and FHV: *P* = 0.86; CI-inducing strains: DCV: *P* = 0.67 and FHV: *P* = 0.71) or as viral titre (Pearson’s correlation test: All strains: DCV: *P* = 0.58 and FHV: *P* = 0.95; CI-inducing strains: DCV: *P* = 0.87 and FHV: *P* = 0.75).

### Antiviral protection is costly

As *Wolbachia* is vertically transmitted, reductions in the survival or fecundity of *Wolbachia*-infected females will reduce the fitness of both the host and the symbiont. To estimate these costs, we measured egg hatch rates (in parallel to the CI crosses, 16,469 eggs from 555 females), early-life fecundity (280,260 eggs from 1,548 females) and female lifespan (913 females) of flies infected with the 16 different *Wolbachia* strains.

We found significant variation in egg hatch rates between fly lines infected with different *Wolbachia* strains (Fig 1C; Deviance = 340,97; df = 16; *P* < 0.0001). When the father was uninfected, four strains caused a significant reduction in hatch rates, with three of them resulting in less than 40% of the eggs hatching (Fig 1C, grey bars). Additionally, when both the mother and father were infected, there was a trend towards even lower hatch rates, with two more strains becoming significant (Fig 1C, blue bars). This suggests that male fertility is also being reduced by *Wolbachia* or that rescue of CI is not perfect for some of the strains (ie the modification of sperm in males that is required for CI still causes embryonic mortality when the egg is infected).

Fecundity and lifespan are also affected by *Wolbachia*. For fecundity, two strains increased and two strains reduced the number of eggs laid (Deviance = 250.55; df = 16; *P* < 0.0001; Fig 1D). *Wolbachia* also affected female survival (Deviance = 52.37; df = 16; *P* < 0.0001), with five of the sixteen strains significantly shortening lifespan (Fig 1E).

**Fig 3:**
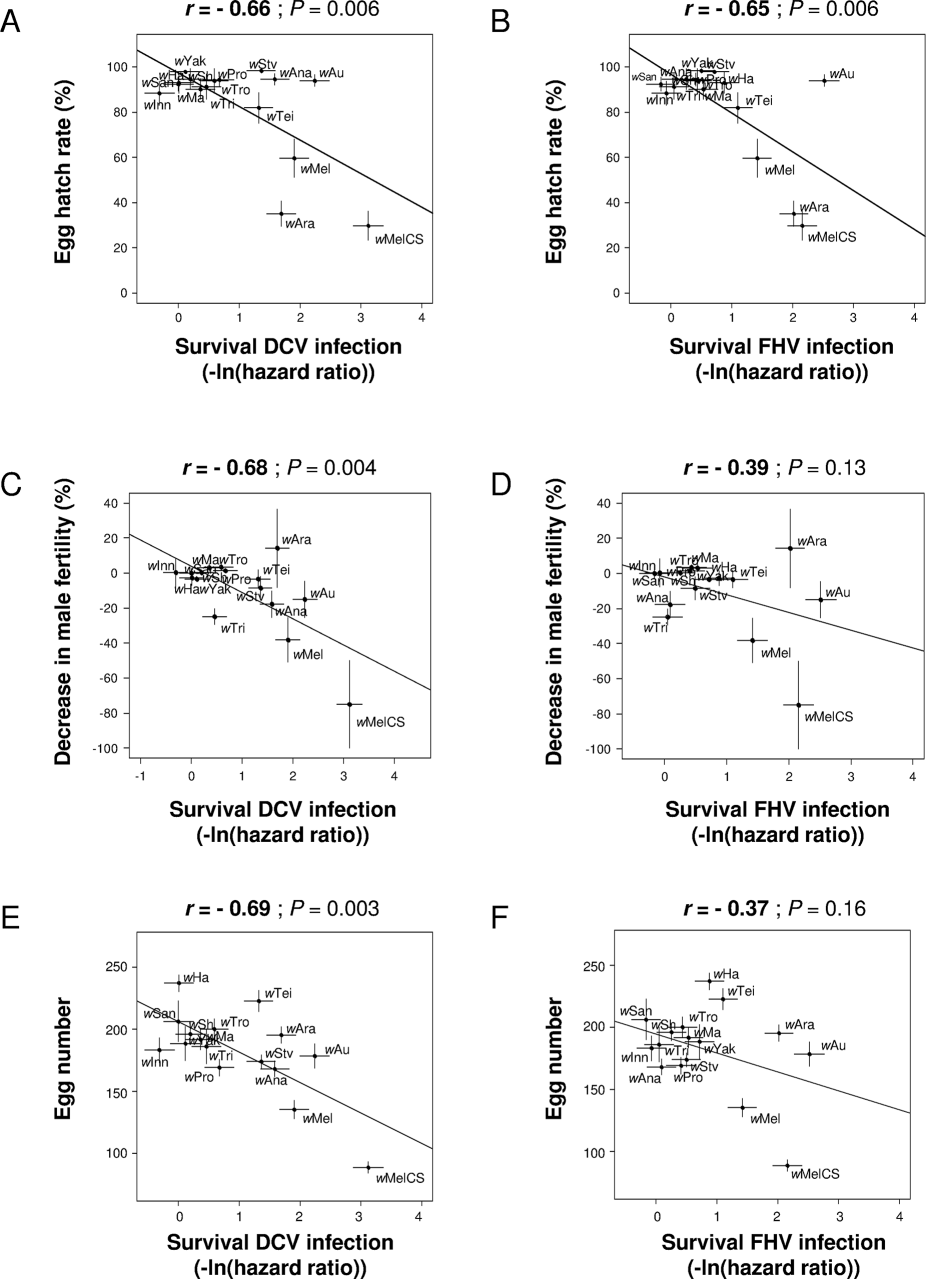
Correlations between antiviral protection and other host life-history traits. A and B: correlation between survival after viral infection and egg hatch rates in crosses between *Wolbachia*-free males and *Wolbachia*-infected females. Virus infections used (A) DCV and (B) FHV [14] (0 and positive values mean no difference and increase in survival compared to *Wolbachia*-free control respectively). C and D: correlation between decrease in male fertility in crosses between *Wolbachia*-infected parents and survival after infection with (C) DCV and (D) FHV. E and F: correlation between egg number and survival after infection with (E) DCV and (F) FHV. Means and standard errors are shown. Solid lines show predicted values from linear regressions. *r* is the Pearson’s correlation coefficient between traits.

The strains that provide the greatest protection against viruses (measured as survival) tended to cause the greatest reductions in the other life-history traits of the flies. Hatch rates of *Wolbachia*-infected females were significantly reduced in flies carrying the symbionts providing the highest levels of protection against both DCV and FHV, whatever the *Wolbachia*-infection status of males (Fig 3A and 3B; S2A and S2B Fig). Because the tested traits are not phylogenetically independent, we reanalyzed these correlations using phylogenetic independent contrasts (see methods). The correlations between hatch rates and level of protection were robust to the phylogenetic non-independence of the data (S1 Table). Higher levels of antiviral protection were also associated with reduced male fertility (Fig 3C and 3D) and lower fecundity (Fig 3E and 3F), but these correlations were only significant in case of DCV. Phylogenetic independent contrasts analyses also showed that correlations with male fertility and fecundity were significant but it strongly depended on the branch length used in the linear models (S1 Table). No correlation with the level of protection and female lifespan was detected (S2C and S2D Fig; note the smaller sample sizes for this trait). Interestingly, *w*Au, which is a native strain of *D*. *simulans*, provides high antiviral protection yet induced little reduction in hatch rates or fecundity.

If the antiviral effects of *Wolbachia* were measured as changes in viral titres rather than survival, most of the correlations became non-significant or marginally-significant, but the direction of the relationships remained the same, with low viral titres associated with stronger costs (S3A–S3J Fig). Again, costs induced by *w*Au on hatch rates were generally lower than expected by the correlations with viral titres.

### Cytoplasmic incompatibility is not costly

**Fig 4:**
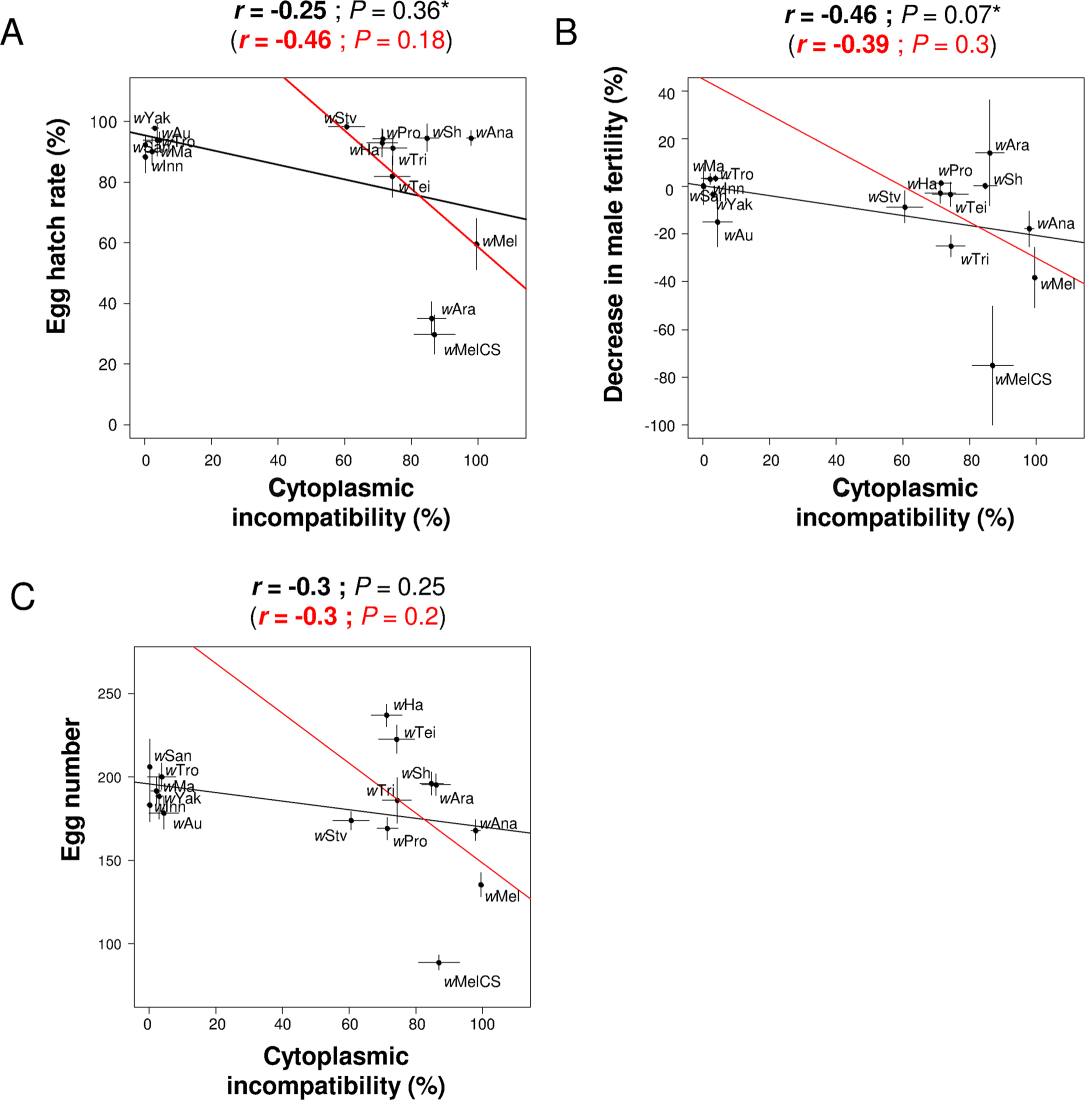
Correlations between CI and other host life-history traits. The level of CI is correlated with (A) the egg hatch rates in crosses with *Wolbachia*-free males, (B) the decrease in male fertility and (C) the egg number. Means and standard errors are shown. Solid lines show predicted values from linear regressions using all strains (black) or only CI-inducing strains (red). *r* is the Pearson’s or Spearman’s (*) correlation coefficient between traits.

Similar to antiviral protection, we tested for correlations between levels of CI and other components of host fitness. There was no significant correlation between the level of CI and male fertility, female fecundity, lifespan or the hatch rate of eggs from crosses between *Wolbachia*-infected females and uninfected males (Fig 4A–4C; S4B Fig). In crosses where both parents were *Wolbachia*-infected, the level of CI was negatively correlated with hatch rates (S4A Fig). This was only the case when both CI inducing and non-CI inducing strains were analyzed, and it may reflect incomplete rescue of cytoplasmic incompatibility.

### *Wolbachia* density mediates the trade-off between protection and cost

We hypothesized that *Wolbachia* must infect the germline to induce CI and somatic tissues to provide antiviral protection, so differences in tissue tropism between symbiont strains may partly explain why they have different phenotypic effects on their hosts. To examine this, we measured *Wolbachia* density in somatic tissues (head and thorax of females), testes and freshly laid eggs (as a proxy for the female germline).

**Fig 5:**
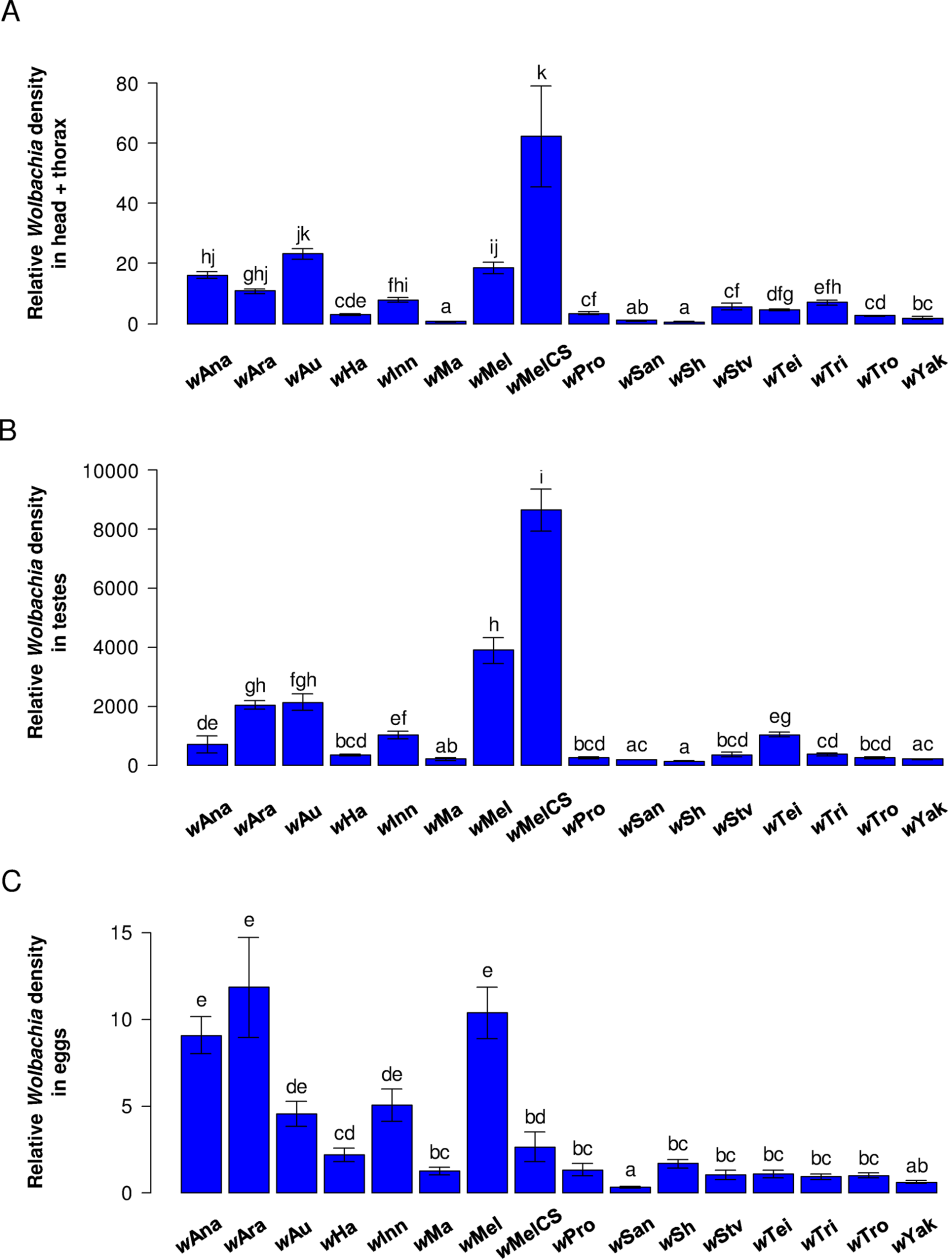
*Wolbachia* tissue tropism. Mean *Wolbachia* density in (A) head and thorax of females, (B) testes and (C) freshly laid eggs. Error bars are standard errors. Letters indicate significant differences based on a Tukey’s honest significance test on ln-transformed data. All tissues were analyzed in a single linear model to test for difference in tissue tropism: strain effect: F_15,427_ = 131. 1; *P* < 0.0001; tissue effect: F_2,427_ = 4448. 8; *P* < 0.0001; strain × tissue effect: F_30,427_ = 11.5; *P* < 0.0001.

There were large between-strain differences in density (Fig 5A–5C). For example, in somatic tissues the *Wolbachia* copy number varies over a 19-fold range. Furthermore, the strains have different tissue tropisms, with a significant strain-by-tissue interaction (Fig 5A–5C). The density in the testes and head + thorax tended to be tightly correlated (Pearson’s correlation test: *r* = 0.89; *P* < 0.0001), and frequently differed from the density in eggs (Pearson’s correlation test: head + thorax–eggs: *r* = 0.63; *P* = 0.01; testes–eggs: *r* = 0.61; *P* = 0.013).

**Fig 6:**
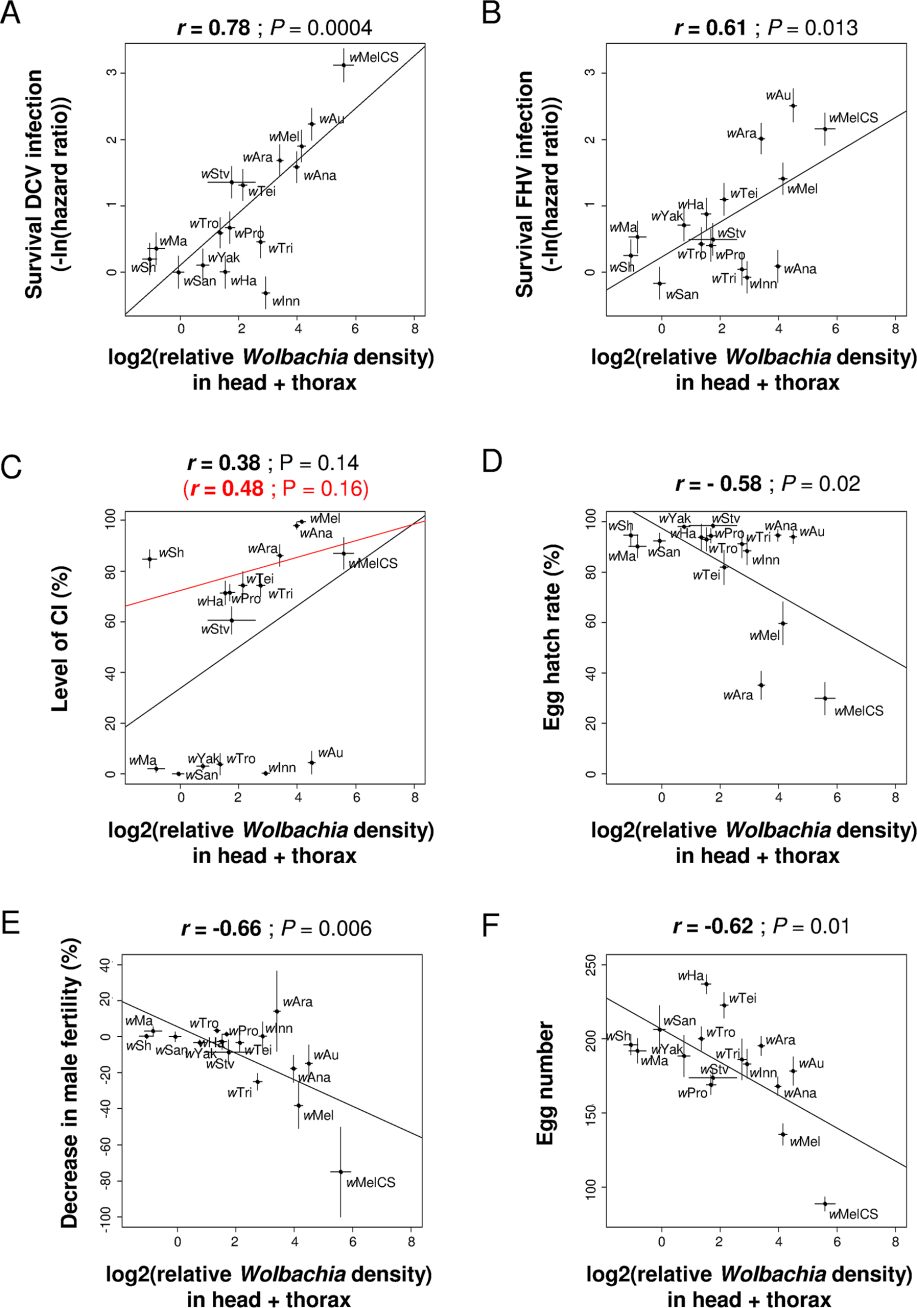
Correlations between *Wolbachia* density in somatic tissues and antiviral protection, CI or other host life-history traits. The relative *Wolbachia* density in head and thorax of females is correlated with survival [14] upon infection with (A) DCV or (B) FHV (0 and positive values mean no difference and increase in survival compared to *Wolbachia*-free control respectively), (C) the level of CI, (D) the egg hatch rate in crosses with *Wolbachia*-free males, (E) the decrease in male fertility and (F) the egg number. Means and standard errors are shown. Solid lines show predicted values from linear regressions. *r* is the Pearson’s correlation coefficient between traits.

Variation in *Wolbachia* density can explain between-strain differences in antiviral protection but not differences in CI. Protection against DCV and FHV was positively correlated with *Wolbachia* density in head and thorax, whether measured as survival (Fig 6A and 6B) or viral titres (S5A and S5B Fig), even after removing potential phylogenetic effects (S1 Table). This holds when both the density in the soma and eggs are included as predictive variables: protection shows a significant partial correlation with density in the soma but not with density in the eggs (only marginally significant for FHV titre; S2 Table). On the contrary, there is no correlation between levels of CI and density in the somatic tissues (Fig 6C), in the testes or in the eggs (S6A–S6B Fig). The only exception to this was when only analyzing CI-inducing strains, levels of CI were positively correlated to the *Wolbachia* density in eggs (red line in S6B Fig; note eggs are uninfected in the CI cross).

The negative effects of *Wolbachia* on host life-history traits are related to the symbiont density, with hatch rates, male fertility and fecundity all negatively correlated to the *Wolbachia* density in the somatic tissues (Fig 6D–6F) but not with the density in the eggs (Pearson’s correlation test: Hatch rate with uninfected father: *P* = 0.08; hatch rate with infected father: *P* = 0.06; male fertility: *P* = 0.58; fecundity: *P* = 0.27). The same conclusion holds when controlling for the *Wolbachia* phylogeny (S1 Table), although for male fertility and fecundity significance depends on the branch length used for the linear model. When these traits are analyzed with a multiple regression, they show significant partial correlations with density in the soma but not with density in the eggs (S2 Table). There was no correlation between female lifespan and *Wolbachia* density in any of the tissues (Pearson’s correlation test: head + thorax: *P* = 0.73; testes: *P* = 0.32; eggs: *P* = 0.13).

## Discussion

Heritable bacterial symbionts have successfully colonized a wide range of arthropods by using a diversity of strategies ranging from mutualism to parasitism. Typically the evolution of these symbiont strategies has been considered in isolation, but this can be misleading if there are trade-offs between these traits and other components of host or symbiont fitness. Identifying these trade-offs is not only a prerequisite to understand the evolution of symbiosis, but will also inform the use of symbionts in applied programs. Using a set of *Wolbachia* strains that provide varying levels of protection against viral pathogens, we found that this mutualistic effect was independent of the ability to parasitically manipulate host reproduction. Antiviral protection relies on the bacteria reaching high densities in somatic tissues and is associated with strong reductions in several host life-history traits, while reproductive parasitism is not linked to symbiont density in somatic tissues and not costly to infected females.

While some symbionts are mutualists that spread through populations by increasing host fitness and others are parasites that manipulate host reproduction, others simultaneously have both effects [11]. It is already well known that in *Wolbachia* antiviral protection and CI are highly genetically variable traits [14,27,37,44]. However, to our knowledge, our study is the first to assess both traits in a wide array of strains in a common host genetic background. We found no correlation between the expressions of these phenotypes, with four strains only providing protection, two strains only inducing CI, eight strains inducing both protection and CI, and two strains showing neither phenotype. Therefore, these traits have independent evolutionary trajectories. Some strains may also rely on alternative strategies to be maintained in populations, such as enhancing the host fecundity or other fitness components [45]. For instance, two of the tested strains in our study were associated with increased fecundity.

Besides antiviral protection and reproductive manipulation, *Wolbachia* infections can induce fitness costs, with important life-history traits being affected such as lifespan, fecundity, egg viability or larval development and competitiveness [30,46–53]. In accordance with previous studies, we found *Wolbachia*-induced costs on several traits that should reduce both the fitness of the host and of *Wolbachia*. In some cases these costs could be very large–for example some strains result in the majority of infected eggs never hatching, suggesting that those strains might not be able to invade natural host populations.

We found that antiviral protection trade-offs with egg hatch rates, female fecundity and male fertility. In many cases highly protective strains induced substantial reductions in these fitness components. Because *Wolbachia* relies on host reproduction for its transmission, these trade-offs will affect both the host and symbiont, as both partners benefit from antiviral protection and both will suffer from reduced female reproduction. Further evidence that antiviral protection is costly comes from a comparison of the two main *Wolbachia* genotypes in *D*. *melanogaster* populations, which showed that the genotype that provided the greatest antiviral protection also shortened the lifespan of infected flies (Chrostek et al. 2013). Similarly, when *w*Au is transferred into *D*. *melanogaster* it reaches high densities, provides strong protection against viruses and shortens the lifespan of flies (Chrostek et al. 2014). Interestingly, using a similar experimental design to ours, another study showed that high levels of protection conferred by the symbiont *Hamiltonella defensa* against parasitoids in aphids are associated with less costly symbiont strains contrary to what we found [54]. While the mechanisms of protection in *Wolbachia* remain to be elucidated, in *H*. *defensa* it is known that protection relies on the presence of a bacteriophage encoding a toxin [55,56]. It is likely that different mechanisms of protection lead to different trade-offs with host life-history traits.

The reason that *Wolbachia*-mediated antiviral protection is so costly appears to be that it requires high symbiont densities. The density of *Wolbachia* in host tissues have been repeatedly shown to be involved in the ability of the bacteria to protect against viruses [12,14,27,36–38,57,58], and this was also the case in the present study with high protection being associated with higher densities in the somatic tissues of the flies. Using our sixteen *Wolbachia* strains we were able to test for a correlation between density and costs, and found that high densities of the bacteria in somatic tissues correlate with lower egg hatch rates, male fertility and fecundity. Harboring high loads of *Wolbachia* might be harming flies due to a re-allocation of resources from host to symbiont or pathological effects of the symbiont infection. Accordingly, *w*MelPop, a mutant strain that over-replicates causes a severe life-shortening effect [38,48,59] and other high density *Wolbachia* genotypes in *D*.*melanogaster* are associated with reduced lifespan [27,34]. The correlations between antiviral protection, costs on life-history traits and *Wolbachia* density remained when controlling for phylogenetic effects, which supports the hypothesis that there is a causal link between antiviral protection and costs that is mediated by symbiont density.

Contrary to antiviral protection, we did not observe any trade-off between the expression of CI and the other host fitness components. The explanation for this is likely that CI levels were not correlated to the density of *Wolbachia* in somatic tissues (note that our sample size is limited if considering just CI inducing strains). CI is thought to be the result of a sperm modification causing improper segregation of the paternal chromosomes after fertilization of the egg [60]. Rather than the overall density of *Wolbachia* in the somatic tissues, it is the ability of the bacteria to specifically colonize sperm cysts that is thought to allow the expression of CI [39,42]. For this reason we investigated whether differences in tissue tropism between strains might affect whether they cause CI. While tissue tropism did vary, there was no correlation between density in testes and CI, but it may be that this is a poor proxy for the number of sperm cysts that are infected. However, we found that, among CI-inducing strains, levels of CI were positively correlated with the bacterial density in eggs (our measure of female germline density), similar to what was found in another study [40]. It is possible that higher density in the eggs might correlate with bacteria targeting the germ line in developing male embryos. Alternatively, the egg is the site of the rescue activity that prevents the expression of CI in *Wolbachia*-infected embryos [60], so strains inducing high levels of CI may have evolved towards higher density in the egg to overcome the effect of the sperm modification.

Our findings have important implications regarding the evolution of *Wolbachia* symbioses, as trade-offs will act as a constraint on the evolution of mutualism (protection) but not reproductive parasitism (CI). Selection will act on both host and parasite genes to reduce the cost of *Wolbachia* infection, and alone this is likely to lead to the evolution of lower bacterial densities and therefore reduced antiviral protection. Thus, unless antiviral protection is sufficiently strongly selected for, it may reach lower levels or even disappear as the two partners coevolved towards less harmful *Wolbachia* infection. This prediction is supported by the partial replacement of the highly protective strain *w*MelCS by *w*Mel, a lower density strain inducing lower protection, in populations of *D*. *melanogaster* [25–27]. Strikingly, in the pathogenic strain *w*MelPop, the symbiont density and the associated level of protection and costs on other life-history traits have been shown to evolve quickly, over a few host generations, suggesting that such changes may rapidly occur in nature [38]. As the most protective strains are very costly, they may only be favoured when there is very strong selection by viruses.

Over the long term, selection may sometimes be able to break a trade-off [61] and lead to the evolution of *Wolbachia* strains that provide the benefits of antiviral protection but without the associated costs. Because we transferred most of the symbiont strains from other species into *D*. *simulans*, the control of the bacterial density and associated costs is expected to be inefficient due to a lack of coevolution between the two partners. This situation therefore reflects new associations that have arisen by horizontal transmission (as frequently occurs during the evolution of *Wolbachia*). We had one highly protective strain that naturally occurs in *D*. *simulans*, and this strain induced little cost on egg hatch rates despite showing rather high bacterial density and strong protection. This strain does not induce CI and yet shows rapid spread in natural populations [20,21] suggesting that protection might be the selective force driving the evolution of this strain. While this suggests that natural selection may be able to break the association between antiviral protection and cost, this may not be inevitable as naturally occurring protective *Wolbachia* strains in *D*. *melanogaster* still reduce the lifespan of flies [27].

Whether CI or antiviral protection is favored by selection will depend not only on the costs of these traits but also the strength of selection favouring the trait. Selection on the symbiont to evolve CI may often be very weak–there is no selection for the phenotype in males in panmictic populations [32,62], and its evolution relies on population structure generating local relatedness [63] [64] (see [65] for an alternative explanation). Our observation that CI is not associated with costly changes in the phenotype of infected females (the transmitting sex) means there may often be little selection on the symbiont to reduce the strength of CI, making it stable over evolutionary time even when population structure is weak.

Finally, our results have implications for the control of vector-borne viral diseases by the introduction of *Wolbachia* into mosquito populations, as such efforts may fail if selection to reduce the cost of infection leads to reduced symbiont density and therefore the loss of antiviral protection [66]. This is even more likely if viruses cause little harm to its vector or are rare in the vector population, thus inducing little selective pressure on protection [23]. This is the case for the main target of these control efforts, dengue virus, which is thought to only decrease the fitness of mosquitoes by a few percent [67] and its prevalence in mosquito populations is low [68]. Therefore, the long-term maintenance of protection may rely on selection by the wider community of viruses favouring protection. The first releases of *Wolbachia* infected *Aedes aegypti* mosquitoes took place in 2011 [29], and one year later the *Wolbachia* strain still protected against dengue virus infection [31]. Only further monitoring over future years will determine whether this is truly an ‘evolution proof’ method of disease control.

## Methods

### *Drosophila* lines and *Wolbachia* strains

All *Wolbachia* strains were in the *D*. *simulans* STCP line that was generated by six generations of sib matings [69]. *Wolbachia* was previously backcrossed or microinjected into the STCP line [14,44,69,70].

Flies were maintained on a cornmeal diet at 25°C, 12 hours light/dark and 70% relative humidity. To minimize inbreeding effects, before each experiment STCP females were crossed to males of a different *Wolbachia*-free isofemale line (14021–0251.175, Dsim\wild-type, San Diego *Drosophila* Species Stock Center). Groups of 30 first instar F1 larvae were then transferred to new vials to ensure a constant larval density. Measurements of fitness traits were carried out on emerging F1 adults. Except for the fecundity measurements, F1 larvae were raised on a standard cornmeal diet (agar: 1%, dextrose: 8.75%, maize: 8.75%, yeast: 2%, nipagin: 3%) with 100 μl of 15% liquid yeast on the top of the food. For the fecundity experiment (see below), F1 larvae developed on a diet depleted in maize (4.4%) and dextrose (4.4%) with no added yeast to create less favorable conditions. Two generations before the experiments, *Wolbachia* infection statuses were checked by PCR using primers wsp81F and wsp691R [71].

### Hatch rates and cytoplasmic incompatibility

Virgin F1 male and female flies were collected and aged for 3 and 5 days respectively. Because multiple male matings can decrease the strength of CI [72,73], a male and female were placed in a vial for 4–8 hours. In *D*. *simulans*, remating does not occur within 8 hours after the first copulation (Nina Wedell, personal communication). Females were then placed individually on a 50 mm diameter Petri dish with standard cornmeal diet containing food coloring with 15 μl of 15% liquid yeast on the top of the food. Around 20 hours later, females were removed and eggs were counted. Females that laid five or less eggs were discarded. Hatch rates were estimated by counting unhatched eggs about 35 hours later. The compatible crosses between uninfected males and uninfected females showed a mean egg hatch rate of 98%, thus suggesting that most females in this experiment were mated. Moreover, for *Wolbachia*-infected lines, discarding potentially non-mated females for which none of the eggs hatched did not change the significance of correlations with the other traits as mean hatch rates with or without those females were strongly correlated (Pearson’s correlation test: r ≈ 0.99, df = 14, *P* < 0.0001).

### Fecundity

F1 larvae were raised on our poor diet, and 0 to 2-day-old flies were placed on standard cornmeal food with live yeast on the surface to stimulate egg maturation. After 2 days (2- to 4-day-old), 3 males and 3 females were placed in petri dishes of colored poor diet. Over 6 days, flies were anaesthetized with CO_2_ and transferred onto a new dish every 24 hours. The number of eggs was recorded by photographing the Petri dish and counting eggs using a multi-point counter tool in ImageJ [74].

### Female lifespan

As for the hatch rate experiment, F1 larvae were raised on our standard cornmeal diet. Five male and 5 female freshly emerged flies were placed per vial on poor diet. Flies were tipped onto fresh food every 3 days and the number of dead female flies recorded daily for 72 days until all flies died.

### Dissections

To investigate *Wolbachia* tissue tropism, F1 larvae were reared on standard diet, and virgin males and females aged to 3 and 5-day-old respectively. Males were then anaesthetized on ice and dissected in Ringer’s solution [75]. For each *Wolbachia* strain, 10 pools of 5 pairs of testes were collected.

Five-day-old females were allowed to mate with 2- to 4-day-old virgin *Wolbachia*-free STCP males for 24 hours. Females were then isolated and 10 replicates of 3 females per strain were placed in Petri dishes onto grape agar food with 15 μl of 15% liquid yeast on the top. After 6 to 8 hours, 20 eggs were harvested from each Petri dish and transferred into a microcentrifuge tube. In parallel, the head and thorax was separated from the abdomen of 6-day-old females. For each *Wolbachia* strain, 10 replicates, each consisting of a pool of head and thorax collected from 10 females were transferred into microcentrifuge tubes. All tissues were frozen at -80°C for DNA extraction.

### DNA extraction and quantitative PCR

DNA was extracted from the tissue samples using EconoSpin All-In-One Silica Membrane Mini Spin Columns (Epoch Biolabs) and the QIAamp DNA Micro kit (Qiagen). Using the extracted DNA, quantitative PCR (qPCR) was used to determine the *Wolbachia* density in the carcasses (head and thorax), testes and eggs. For carcasses and testes, the amount of the *Wolbachia* gene *atpD* (atpDQALL_F: 5’-CCTTATCTTAAAGGAGGAAA-3’; atpDQALL_R: 5’-AATCCTTTATGAGCTTTTGC-3’) relative to the endogenous control gene *actin 5C* (Forward primer: 5’-GACGAAGAAGTTGCTGCTCTGGTTG-3’; Reverse primer: 5’-TGAGGATACCACGCTTGCTCTGC-3’) was quantified using the SensiFAST SYBR & Fluorescein kit (Bioline). The *Wolbachia* density was estimated as: *2* ^*ΔCt*^, where *Ct* is the cycle threshold and *ΔC t = Ct*_*actin5C*_ *-Ct*_*atpD*_. The PCR cycle was 95°C for 2 min, followed by 40 cycles of 95°C for 5 s, 55°C for 10 s, 72°C for 5 s. Since embryo mortality due to *Wolbachia* was observed in our experiment on hatch rate, the *Wolbachia* density in eggs was estimated as the amount of the gene *atpD* in a sample relative to the amount of the same gene in a positive control placed on every qPCR plate as follow: *2* ^*ΔCt*^, where *Ct* is the cycle threshold and *ΔC t = Ct*_*positive control*_ *-Ct*_*atpD*_. For each sample, two qPCR reactions (technical replicates) were carried out and a linear model was used to correct for plate effects.

### Statistical analysis

Statistical analyses were performed using R [76]. Hatch rates were analyzed using mixed effect generalized linear models with a logit link function and the effect of individual mothers treated as random (package lme4). Fecundity was analyzed using a linear model with the total number of eggs laid over 6 days as a response and a random temporal block effect. Female lifespan was analyzed with a generalized linear model with the *Wolbachia* infection status as a fixed effect and vial as a random effect. To test the effects of *Wolbachia* strain individually on these traits we performed multiple comparisons with the control cross (uninfected flies) using Dunnett’s test (package multcomp).

*Wolbachia* densities within tissues were log2-transformed and analyzed with a linear model including the effect of the *Wolbachia* strain, tissue, and their interaction. Between-strain differences in density were tested using multiple comparisons (Tukey’s HSD test, package multcomp).

Between-trait correlations were tested with Pearson’s correlation tests unless the assumptions of normality and homoscedasticity were not reached, in which case Spearman’s tests were used. In order to take into account the phylogenetic non-independence of the data, significant correlations were further analyzed using independent contrasts [77] with the function crunch (R package caper) [78] (See S1 Table).

## Supporting Information

S1 Fig Relationship between CI and viral titres. The levels of CI is estimated as the percentage of unhatched eggs relative to the mean hatch rate in crosses between uninfected females and uninfected males. The titre of (A) DCV and (B) FHV was measured in [14]. Means and standard errors are shown. Solid lines show predicted values from linear regressions using all strains (black) or only CI-inducing strains (red). *r* is the Pearson’s correlation coefficient between traits.
(TIF)

S2 Fig Correlations between antiviral protection and other host life-history traits. Correlation between egg hatch rates in crosses with *Wolbachia*-infected males and level of protection measured as survival upon infection with (A) DCV and (B) FHV (0 and positive values mean no difference and increase in survival compared to *Wolbachia*-free control respectively). Correlation between female lifespan and level of protection upon infection with (C) DCV and (D) FHV. Means and standard errors are shown. Solid lines show predicted values from linear regressions. *r* is Pearson’s the correlation coefficient between traits.
(TIF)

S3 Fig Correlations between viral titres and host life-history traits. Correlations between DCV titre measured in [14] and egg hatch rates in crosses with (A) *Wolbachia*-free males or (B) *Wolbachia*-infected males, (C) decrease in male fertility, (D) egg number and (E) female lifespan. Correlations between FHV titre measured in [14] and egg hatch rates in crosses with (F) *Wolbachia*-free males or (G) *Wolbachia*-infected males, (H) decrease in male fertility, (I) egg number and (J) female lifespan. Means and standard errors are shown. Solid lines show predicted values from linear regressions. *r* is the Pearson’s correlation coefficient between traits.
(TIF)

S4 Fig Correlations between CI and other host life-history traits. (A) Correlation between egg hatch rates in crosses with *Wolbachia*-infected males and level of CI. (B) Correlation between female lifespan and level of CI. Means and standard errors are shown. Solid lines show predicted values from linear regressions using all strains (black) or only CI-inducing strains (red). *r* is the Pearson’s or Spearman’s (*) correlation coefficient between traits.
(TIF)

S5 Fig Correlations between somatic *Wolbachia* density and viral titres. Relative *Wolbachia* density in head and thorax of females is correlated with viral titre [14] upon infection with (A) DCV or (B) FHV. Means and standard errors are shown. Solid lines show predicted values from linear regressions. *r* is the Pearson’s correlation coefficient between traits.
(TIF)

S6 Fig Correlations between *Wolbachia* density, CI and other host life-history traits. (A) Relative *Wolbachia* density in testes and level of CI. (B) Relative *Wolbachia* density in freshly laid eggs and level of CI. (C) Relative *Wolbachia* density in head and thorax of females and egg hatch rate in crosses with *Wolbachia*-infected males and females. Means and standard errors are shown. Solid lines show predicted values from linear regressions using all strains (black) or only CI-inducing strains (red). *r* is the Pearson’s correlation coefficient between traits.
(TIF)

S1 Table Independent contrasts analyses of between-trait correlations. (DOCX)

S2 Table Partial correlations between antiviral protection or other host fitness components with *Wolbachia* density in somatic tissues or the female germline. (DOCX)
