# Supplementary figures and images for: Should Symbionts Be Nice or Selfish? Antiviral Effects of Wolbachia Are Costly but Reproductive Parasitism Is Not
Source: PLoS Pathog. 2015 Jul 1;11(7):e1005021. doi: 10.1371/journal.ppat.1005021 (PMC4488530; doi:10.1371/journal.ppat.1005021)

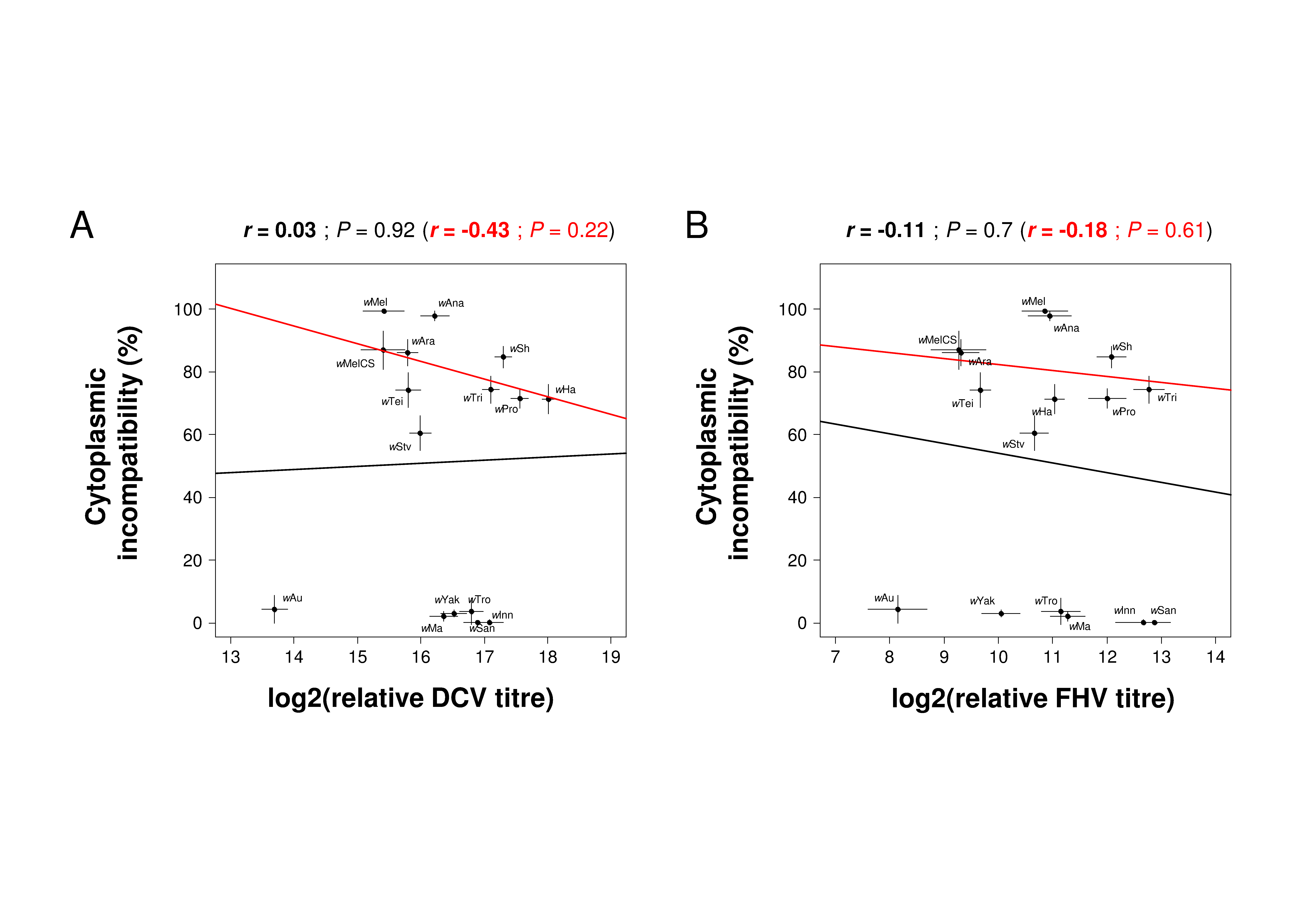

Supplement: S1 Fig — The levels of CI is estimated as the percentage of unhatched eggs relative to the mean hatch rate in crosses between uninfected females and uninfected males. The titre of (A) DCV and (B) FHV was measured in [14]. Means and standard errors are shown. Solid lines show predicted values from linear regressions using all strains (black) or only CI-inducing strains (red). r is the Pearson’s correlation coefficient between traits. (TIF) [file ppat.1005021.s001.tif]

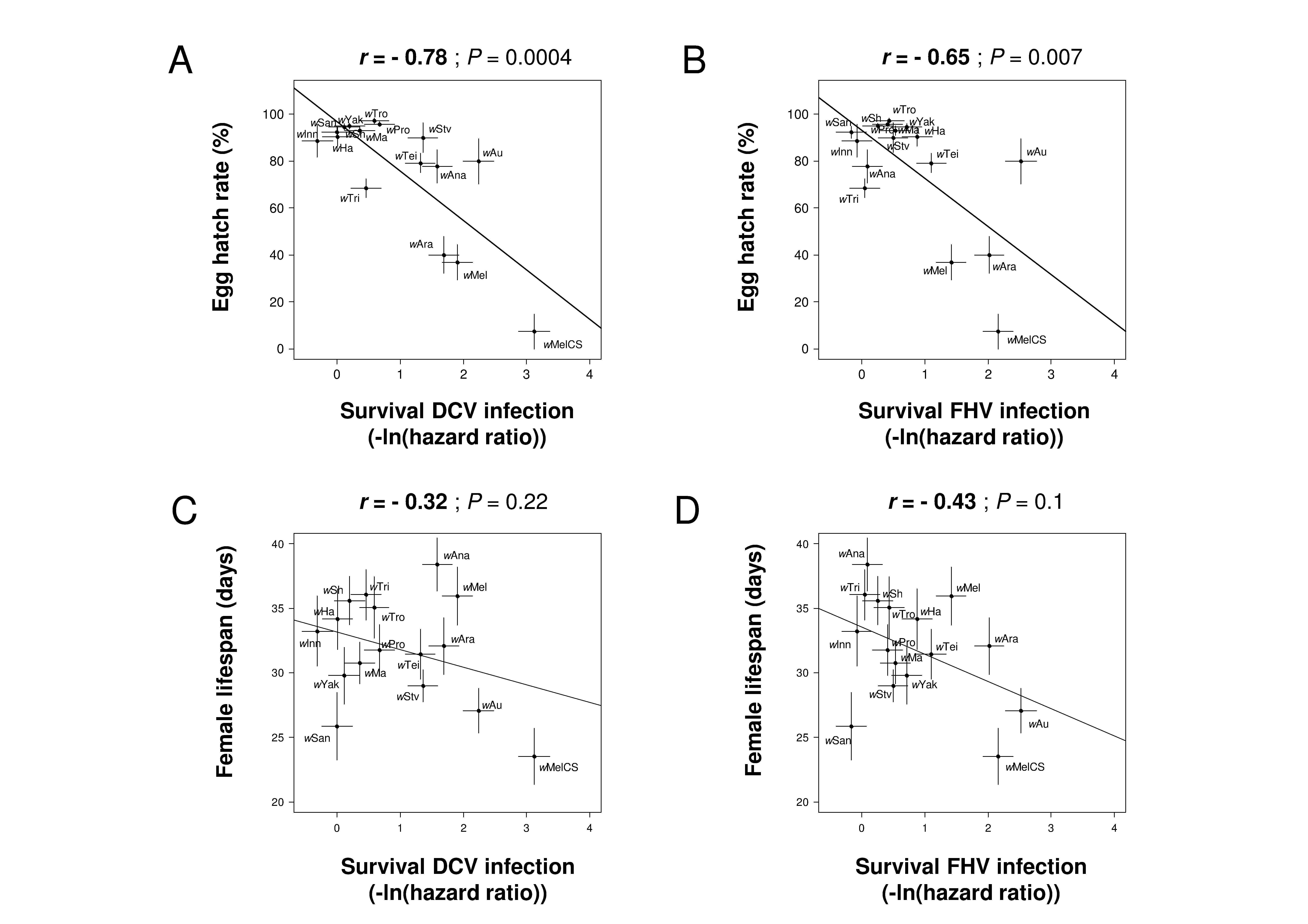

Supplement: S2 Fig — Correlation between egg hatch rates in crosses with Wolbachia-infected males and level of protection measured as survival upon infection with (A) DCV and (B) FHV (0 and positive values mean no difference and increase in survival compared to Wolbachia-free control respectively). Correlation between female lifespan and level of protection upon infection with (C) DCV and (D) FHV. Means and standard errors are shown. Solid lines show predicted values from linear regressions. r is Pearson’s the correlation coefficient between traits. (TIF) [file ppat.1005021.s002.tif]

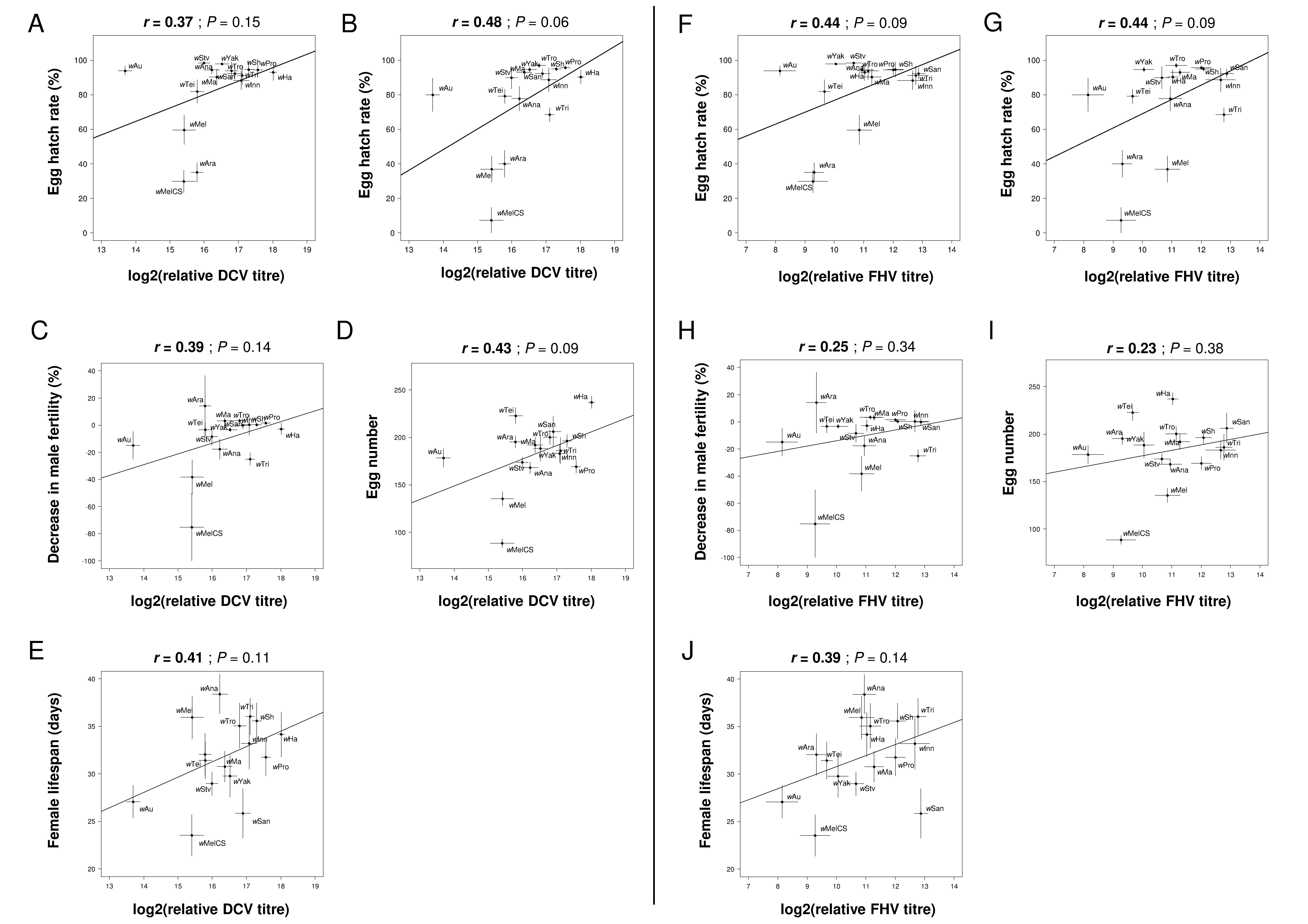

Supplement: S3 Fig — Correlations between DCV titre measured in [14] and egg hatch rates in crosses with (A) Wolbachia-free males or (B) Wolbachia-infected males, (C) decrease in male fertility, (D) egg number and (E) female lifespan. Correlations between FHV titre measured in [14] and egg hatch rates in crosses with (F) Wolbachia-free males or (G) Wolbachia-infected males, (H) decrease in male fertility, (I) egg number and (J) female lifespan. Means and standard errors are shown. Solid lines show predicted values from linear regressions. r is the Pearson’s correlation coefficient between traits. (TIF) [file ppat.1005021.s003.tif]

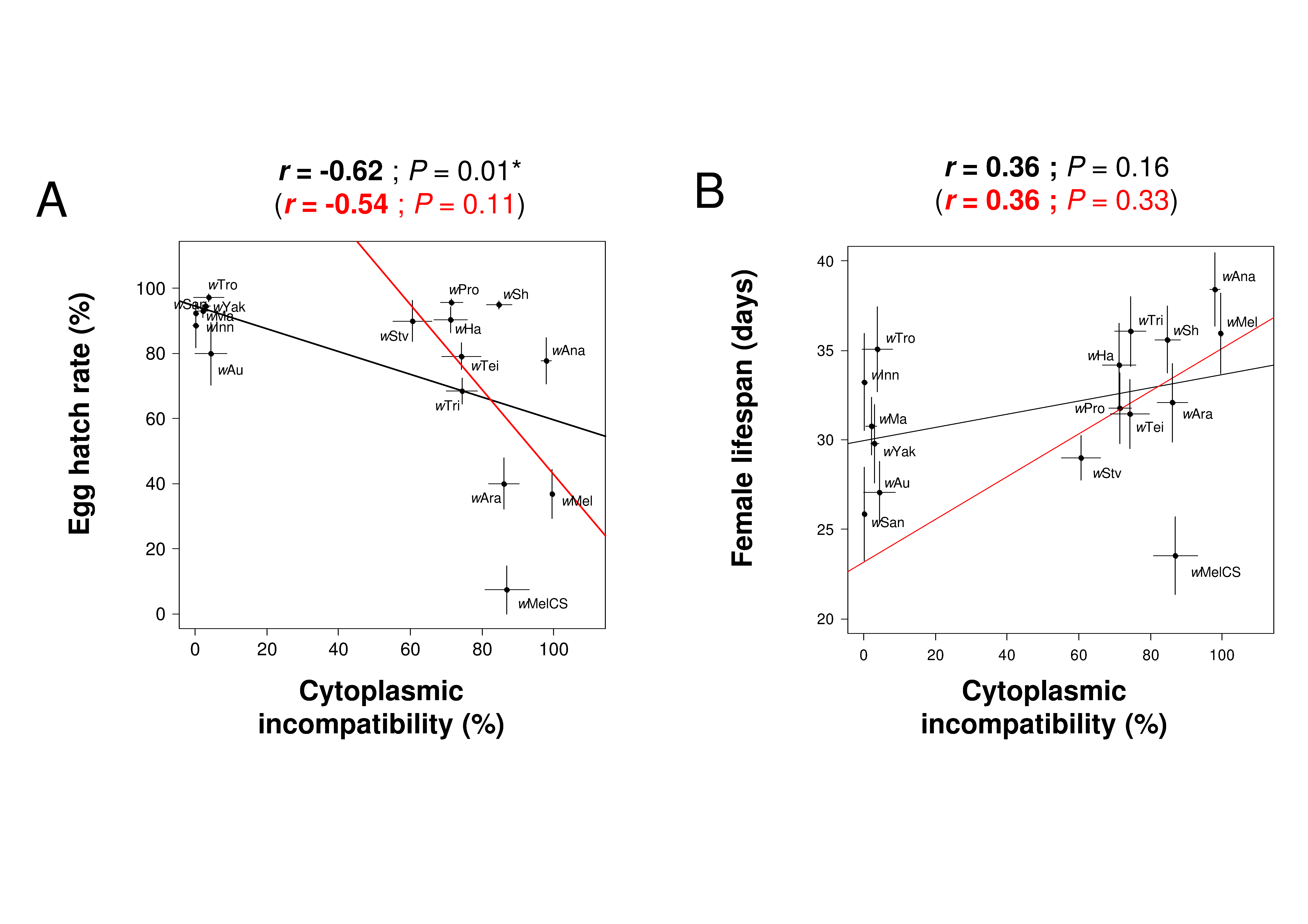

Supplement: S4 Fig — (A) Correlation between egg hatch rates in crosses with Wolbachia-infected males and level of CI. (B) Correlation between female lifespan and level of CI. Means and standard errors are shown. Solid lines show predicted values from linear regressions using all strains (black) or only CI-inducing strains (red). r is the Pearson’s or Spearman’s (*) correlation coefficient between traits. (TIF) [file ppat.1005021.s004.tif]

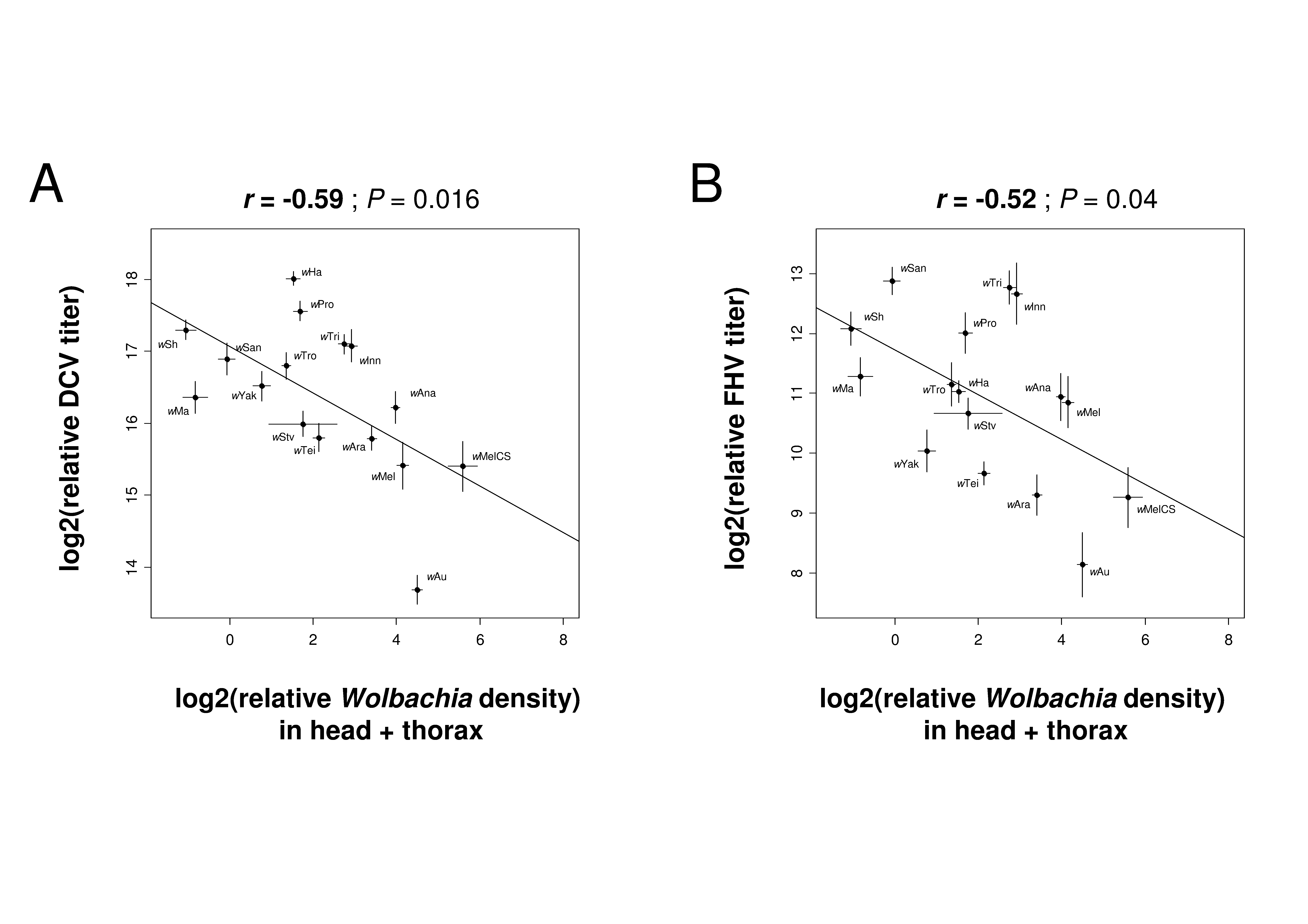

Supplement: S5 Fig — Relative Wolbachia density in head and thorax of females is correlated with viral titre [14] upon infection with (A) DCV or (B) FHV. Means and standard errors are shown. Solid lines show predicted values from linear regressions. r is the Pearson’s correlation coefficient between traits. (TIF) [file ppat.1005021.s005.tif]
