# Supplementary material for: Should Symbionts Be Nice or Selfish? Antiviral Effects of Wolbachia Are Costly but Reproductive Parasitism Is Not
Source: PLoS Pathog. 2015 Jul 1;11(7):e1005021. doi: 10.1371/journal.ppat.1005021 (PMC4488530; doi:10.1371/journal.ppat.1005021)
Supplement: S1 Table — (DOCX) [file ppat.1005021.s007.docx]

| Independent contrasts model | Original branch length | |  | Equal branch length | |
| --- | --- | --- | --- | --- | --- |
|  | *F* statistics | *P* |  | *F* statistics | *P* |
| Egg hatch rate_(uninfected father)_ ~ Protection against DCV | *F_1,11_* = 12.66 | 0.004** |  | *F_1,11_* = 14.02 | 0.003** |
| Egg hatch rate_(uninfected father)_ ~ Protection against FHV | *F_1,10_* = 27.59 | 0.0004*** |  | *F_1,10_* = 35.28 | 0.0001*** |
| Egg hatch rate_(infected father)_ ~ Protection against DCV | *F_1,11_* = 53.4 | < 0.0001*** | | *F_1,11_* = 23.83 | 0.0005*** |
| Egg hatch rate_(infected father)_ ~ Protection against FHV | *F_1,11_* = 13.39 | 0.004** |  | *F_1,11_* = 17.65 | 0.001** |
| Male fertility ~ Protection against DCV | *F_1,10_* = 2.08 | 0.18 |  | *F_1,11_* = 8.84 | 0.01* |
| Male fertility ~ Protection against FHV | *F_1,11_* = 9.13 | 0.01* |  | *F_1,11_* = 4.78 | 0.05* |
| Fecundity ~ Protection against DCV | *F_1,10_* = 27.46 | 0.0004*** |  | *F_1,11_* = 3.9 | 0.07 |
| Fecundity ~ Protection against FHV | *F_1,10_* = 6.129 | 0.03* |  | *F_1,11_* = 0.91 | 0.36 |
|  |  |  |  |  |  |
| Protection against DCV ~ *Wolbachia* density_head + thorax_ | *F_1,11_* = 90.55 | < 0.0001*** | | *F_1,11_* = 23.17 | 0.0005*** |
| Protection against FHV ~ *Wolbachia* density_head + thorax_ | *F_1,11_* = 42.22 | < 0.0001*** | | *F_1,11_* = 15.26 | 0.002** |
| DCV titre ~ *Wolbachia* density_head + thorax_ | *F_1,11_* = 24.39 | 0.0004*** |  | *F_1,11_* = 7.81 | 0.02* |
| FHV titre ~ *Wolbachia* density_head + thorax_ | *F_1,11_* = 28.64 | 0.0002*** |  | *F_1,11_* = 13.15 | 0.004** |
| Egg hatch rate_(uninfected father)_ ~ *Wolbachia* density_head + thorax_ | *F_1,11_* = 8.59 | 0.01* |  | *F_1,11_* = 6.08 | 0.03* |
| Egg hatch rate_(infected father)_ ~ *Wolbachia* density_head + thorax_ | *F_1,11_* = 14.27 | 0.003** |  | *F_1,11_* = 16.76 | 0.002** |
| Male fertility ~ *Wolbachia* density_head + thorax_ | *F_1,10_* = 4.57 | 0.058 |  | *F_1,11_* = 10.86 | 0.007** |
| Fecundity ~ *Wolbachia* density_head + thorax_ | *F_1,10_* = 14.46 | 0.003** |  | *F_1,11_* = 14.46 | 0.15 |

Phylogenetically independent contrasts under a Brownian model of evolution were inferred using Pagel’s method to calculate contrasts at polytomies [1]. Linear models were then used to test for evolutionary relationships between traits. Diagnostic regression tests for the robustness of a contrast model were applied using the function caic.diagnostic to examine the behaviour of the absolute standardised contrasts with the scale of the nodal values, standard deviation at nodes and age of the nodes [2,3]. Since diagnostic tests showed that in all of our models, the assumption of evolution under Brownian motion was violated, we did a second analysis after setting the branch length of the *Wolbachia* phylogeny to equal length in order to reach the Brownian assumption (Table S1). Finally, when contrasts showed studentized residuals > 3, such contrasts were removed from the analysis as commonly applied elsewhere [2,4]. *: *P*<0.05; **: *P*<0.01; ***: *P*<0.001.

1. Pagel M (1992) A method for the analysis of comparative data. J Theor Biol 156: 431–442.

2. Garland TJ, Harvey P, Ives A (1992) Procedures for the analysis of comparative data using phylogenetically independent contrasts. Syst Biol 41: 18–32.

3. Purvis A, Rambaut A (1995) Comparative Analysis by Independent Contrasts (CAIC) User’s Guide.

4. Jones KE, Purvis A (1997) An optimum body size for mammals? Comparative evidence from bats. Funct Ecol 11: 751–756.
