# Supplementary material for: Should Symbionts Be Nice or Selfish? Antiviral Effects of Wolbachia Are Costly but Reproductive Parasitism Is Not
Source: PLoS Pathog. 2015 Jul 1;11(7):e1005021. doi: 10.1371/journal.ppat.1005021 (PMC4488530; doi:10.1371/journal.ppat.1005021)
Supplement: S2 Table — (DOCX) [file ppat.1005021.s008.docx]

| Traits^†^ | | Tissue | Partial correlation coefficient | *P* |
| --- | --- | --- | --- | --- |
| Protection against DCV | Survival | head + thorax | *r* = 0.71 | 0.0003*** |
|  |  | eggs | *r* = -0.07 | 0.79 |
|  | virus titer | head + thorax | *r* = -0.49 | 0.04* |
|  |  | eggs | *r* = -0.02 | 0.94 |
| Protection against FHV | Survival | head + thorax | *r* = 0.47 | 0.05* |
|  |  | eggs | *r* = 0.1 | 0.71 |
|  | virus titer | head + thorax | *r* = -0.41 | 0.1 |
|  |  | eggs | *r* = -0.04 | 0.88 |
| Egg hatch rate | uninfected father | head + thorax | *r* = -0.43 | 0.09 |
|  |  | eggs | *r* = -0.14 | 0.61 |
|  | infected father | head + thorax | *r* = -0.64 | 0.002** |
|  |  | eggs | *r* = -0.02 | 0.93 |
| Male fertility |  | head + thorax | *r* = -0.72 | 0.0001*** |
|  |  | eggs | *r* = 0.44 | 0.08 |
| Fecundity |  | head + thorax | *r* = -0.59 | 0.009** |
|  |  | eggs | *r* = 0.16 | 0.57 |

^†^*Wolbachia* density in testes was not included because it was strongly correlated with somatic density.
